# Long-Term Complications of Open and Robot-Assisted Laparoscopic Radical Prostatectomy in an Afro-Caribbean Population

**DOI:** 10.7759/cureus.25370

**Published:** 2022-05-26

**Authors:** Lakshay Khosla, Jacob N Bamberger, Nayeem Uddin, Gabriel Vizgan, Lauren E Fink, Andrew G Winer

**Affiliations:** 1 Department of Urology, State University of New York Downstate Health Sciences University, New York, USA

**Keywords:** caribbean region, african continental ancestry group, postoperative complications, prostate neoplasms, robotic surgical procedures, prostatectomy

## Abstract

Background

With the Afro-Caribbean population increasing in the United States, their complication profiles following open (ORP) and robot-assisted laparoscopic (RALP) radical prostatectomy warrants investigation. The purpose of this pilot study was to evaluate differences in long-term complications between ORP and RALP in Afro-Caribbeans.

Methods

A retrospective review of patients undergoing ORP or RALP between April 2010 and August 2019 at an academic medical center and county hospital was conducted. Patients who identified as Afro-Caribbean with complete data were analyzed. Complications were classified using the Clavien-Dindo system. Age, transrectal ultrasound prostate volume, preoperative prostate-specific antigen, Gleason scores, and long-term complications (persisting to at least 18 months postoperatively) were compared between procedures using the Mann-Whitney U test or Fisher’s exact test for statistical significance. Multivariable logistic regression was used to assess the odds of complications.

Results

This study included 53 Afro-Caribbean patients (mean age±SD; 65.9±6.8 years, 30 ORP, and 23 RALP). Patients treated by RALP were younger and had lower Gleason scores. Patients who were treated by RALP had a lower association to having ≥1 complications compared to those treated by ORP (OR=0.28, 95%CI 0.09-0.89, p=0.024). In addition, >60% of complications had a Clavien-Dindo grade≤II for both procedures. RALP resulted in fewer grade II complications compared to ORP (OR=0.25, 95%CI 0.08-0.81, p=0.046).

Conclusions

Treatment of Afro-Caribbeans with RALP allows for fewer complications, especially Clavien-Dindo Grade II complications. While previous investigations show that Black populations experience more complications when treated with ORP or RALP compared to other groups, their complication profile is likely not homogenous when considering their sub-ethnic background and must be investigated to understand optimal interventions for prostate cancer.

## Introduction

Radical prostatectomy (RP) for the treatment of localized prostate cancer carries various risks of complications [1,2]. Traditionally, RP has been performed using an open method (ORP); however, the robot-assisted laparoscopic (RALP) method has become more popular recently due to its minimally invasive nature [3,4]. Surgical complications through both methods are best assessed through a standardized reporting system, such as the one established by the Martin and Clavien-Dindo criteria [5]. The Martin and Clavien-Dindo criteria allowed evaluation of surgical procedures based on grading adverse postoperative events [6]. Once introduced in 1992, it has since been used widely in surgical fields and has strong evidence of validity and applicability worldwide [7].

While studies have investigated differences in standardized complications between ORP and RALP, there is a paucity of data on predominantly Afro-Caribbean men undergoing these procedures. Racial/ethnic disparities in surgical outcomes have produced variable surgical experiences, even under an enhanced recovery program [8]. This is especially pertinent to African descent immigrants, a group whose population rose from 816,000 in 1980 to 4.2 million in 2016 in the United States [9]. With the Afro-Caribbean immigrant population consisting of a significant portion of this group and projected to more than double by 2050 [10], it is imperative to investigate complication profiles on a procedure commonly performed on this sub-ethnic group.

The objective of this pilot study was to evaluate differences in long-term complications between ORP and RALP amongst an Afro-Caribbean patient population. 

## Materials and methods

After Institutional Review Board approval (SUNY Downstate IRB: 1766869-1), a retrospective review of patients undergoing ORP or RALP for treatment of localized prostate cancer between April 2010 and August 2019 at both an academic medical center and county hospital was conducted. The patients were assessed at regular intervals until the completion of their treatment and complication profiles were updated at all follow-ups (usually every two to three months) on an ongoing basis. Long-term complications were considered to be any complication that persisted to at least 18 months postoperatively. Patients that self-identified as Black or of African or Caribbean descent and were born in a Caribbean country were included in the study. Patients who did not meet these inclusion criteria or who had incomplete demographic data entries were excluded. The primary endpoint of the study was to evaluate differences in standardized complications using the Clavien-Dindo scale [6]. Patients were split into two groups, ORP or RALP, based on the procedure performed. 

Patient baseline data including age, comorbidities, and laboratory data, including transrectal ultrasound prostate volume, preoperative prostate-specific antigen values, and prostate biopsy Gleason score, were retrieved. Outcomes and complications (e.g., erectile dysfunction, incontinence, lymphocele, vesicoureteral reflux, urethral stricture) were collected from the patient’s electronic medical record. After data collection, long-term complications (complications persisting for at least 18 months) were standardized for all patients using the Clavien-Dindo scale. We confirm the availability of and access to all original data reported in this study. Since this was a retrospective study and data was deidentified within a database, we were exempt from taking informed consent from analyzed patients. However, all participants provided informed consent while undergoing surgery and we adhered to ethical guidelines under the Declaration of Helsinki and its amendments. 

Baseline, laboratory, and complication parameters were compared between the two groups using the Mann-Whitney U test for continuous variables, reported as mean (standard deviation), and Fisher’s exact test for categorical variables, reported as n (%). Multivariate logistic regression, controlling for factors such as age and Gleason scores, was used to evaluate differences in long-term complications between the two groups, using ORP as a reference group. All analyses were two-tailed with α=0.05. All statistical analyses were performed using SPSS Statistics v. 27.0 (IBM Corp., Armonk, NY). 

## Results

Of the 161 Afro-Caribbean patients in the database, 53 were found to undergo prostatectomy with an average age of 65.9 ± 6.8 years. Of these, 30 underwent ORP and 23 underwent RALP. Patients treated by RALP were younger (62.2 ± 6.1 vs 68.7 ± 6.1 years, p=0.001) and had a lower distribution of Gleason scores (p=0.016). Prostate volume (p = 0.693) and preoperative PSA (p = 0.733) did not differ between the two groups (Table 1). 

**Table 1:** Preoperative Patient Characteristics of Afro-Caribbean Cohort undergoing Radical Prostatectomy ^a ^N represents number of patients; % represents percent of total patients ORP: open radical prostatectomy; RALP: robot-assisted laparoscopic radical prostatectomy; PSA: prostate-specific antigen

Patient Characteristic	ORP	RALP	P
Age (years)	68.7 (6.1)	62.2 (6.1)	0.001
Prostate Volume (mL)	35.8 (11.2)	35.1 (12.0)	0.693
PSA (ng/mL)	12.7 (16.0)	9.9 (5.9)	0.733
Gleason Scores; N (%)^a^			
3+3	9 (30.0)	3 (13.0)	0.016
3+4	5 (16.7)	14 (60.9)	
4+3	12 (40.0)	5 (21.7)	
4+4	2 (6.7)	1 (4.3)	
4+5	1 (3.3)	0 (0)	
5+4	1 (3.3)	0 (0)	

Fewer patients treated with RALP had any complications (at least one complication) compared to those treated with ORP (43.5% vs 73.3%, p=0.027) (Table 2). In addition, most of the noted complications had a Clavien-Dindo classification grade of ≤II for both procedures (60% for RALP and 70% for ORP). RALP patients specifically had significantly fewer Grade II complications (30.4% vs 63.3%, p=0.048). Rates of mixed incontinence (P = 0.715), stress incontinence (p = 0.613), and erectile dysfunction (p=0.057) did not differ between groups. 

**Table 2:** Long Term Complication Rates in Afro-Caribbeans undergoing Radical Prostatectomy ^a^ N represents number of patients; % represents percent of total patients. ^b^ Refers to patients who had at least (≥) one complication. ORP: open radical prostatectomy; RALP: robot-assisted laparoscopic radical prostatectomy

Complications; N (%)^a^	ORP	RALP	P
Any Complications^b^	22 (73.3)	10 (43.5)	0.027
Erectile Dysfunction	16 (53.3)	7 (30.4)	0.057
Stress Incontinence	5 (16.7)	4 (17.4)	0.613
Mixed Incontinence	4 (13.3)	4 (17.4)	0.715
Others (lymphocele, vesicourethral leak, urethral stricture, etc)	8 (26.7)	0 (0)	0.030
Clavien-Dindo Grade; N (%)			
Grade I	4 (13.3)	2 (8.7)	0.687
Grade II	19 (63.3)	7 (30.4)	0.048
Grade III	8 (26.7)	5 (21.7)	0.756
Grade IV	2 (6.7)	1 (4.3)	0.496

There was a lower association of having any (≥1) complications for patients treated by RALP compared to ORP (OR=0.28, 95% CI 0.09-0.89, p=0.024). RALP showed a lower odds of Grade II complications (OR=0.25, 95% CI 0.08-0.81, p=0.046); all other complications did not show any significant differences (Table 3).

**Table 3:** Odds of Long Term Complications of Robot-Assisted Laparoscopic Radical Prostatectomy in Afro-Caribbeans Note: All ORs are in reference to Open radical prostatectomy (OR for Open RP set at 1.00)

Clavien-Dindo Grade	OR (95% CI)	P
Any Grade	0.28 (0.09-0.89)	0.024
Grade I	0.62 (0.10-3.71)	0.685
Grade II	0.25 (0.08-0.81)	0.046
Grade III	0.76 (0.21-2.75)	0.755
Grade IV	0.636 (0.05-7.48)	0.494

## Discussion

While radical prostatectomy (RP) is a standard procedure, there remains a lack of data characterizing complication profiles in specific demographic cohorts [1,2]. Specifically, African descent populations are underrepresented in studies or may be represented as a homogenous group, leading to a lack of understanding of the efficacy of RP modalities. This is a significant consideration because African descent groups have a high prevalence of prostate cancer and specific genetic profiles that make them more susceptible [11,12]. The African descent population consists of various sub-ethnic groups, each with different genetic, environmental, and sociocultural characteristics, and therefore prostate cancer treatments may show different findings when treatment efficacy is analyzed. Since the Afro-Caribbean population in the United States is increasing [10], we aimed to demonstrate the rates of long-term complications of open (ORP) and robot-assisted laparoscopic (RALP) in Afro-Caribbeans. 

Afro-Caribbeans may have the highest prevalence of prostate cancer among the African descent groups, potentially due to specific genetic and lifestyle factors [13]. However, the mortality rates from prostate cancer for Afro-Caribbeans are the lowest amongst African descent cohorts in the United States, suggesting a less severe disease course [14]. This suggests that a greater indication for RP exists for Afro-Caribbeans since cancer is more likely to remain localized. Our data supports this by showing that few patients had high Gleason scores for both groups. 

Patients receiving RALP were younger and had lower Gleason scores than patients receiving ORP. This is likely because older patients tend to have more medical comorbidities, severe prostate cancer, and surgical history (e.g., hostile abdomen in prior surgeries), inclining their Urologist to pursue ORP [15,16]. In addition, ORP is often supplemented with radiation and other therapies for high-risk patients [16]. Nevertheless, the choice of ORP or RALP needs further exploration in the context of age and disease progression, especially with the greater trend towards RALP in recent years [2].

Most complications in the present study were classified as minor Clavien-Dindo Grade I and II for both procedures, which contrasts with previous work that showed higher rates of Grade III and V complications [17]; this difference may be due to the previous study clumping African descent groups as “Blacks” rather than stratifying by specific African descent groups. This suggests that Afro-Caribbeans have a unique complication profile following RP. Similar differences may exist for other sub-ethnic groups and Urologic procedures, which should be studied further. In addition, while an excess of long-term complications following RP in African descent individuals compared to Whites has been demonstrated [14,17], there exists a lack of analysis on how Afro-Caribbeans specifically compare. 

Our study suggests that the choice of RP may affect long-term complications. Less Grade II and III complications have been noted in multiethnic cohorts undergoing RALP compared to ORP [2]. While our Afro-Caribbean cohort was in agreement with fewer grade II complications with RALP, no significant differences were seen for grade III complications, again suggesting a unique complication profile for our cohort. In addition, there were fewer rare complications with RALP for Afro-Caribbeans, which is in agreement with prior multiethnic studies [1,2,16]. However, a larger prospective study is required for further support. Nevertheless, we show that a unique complication profile depending on the sub-ethnic group may provide specific benefits or harms that may not be applicable to other groups. Understanding the complication profile will allow for more informed decision-making on the end of the physician and patient, as well as set expectations of management of post-RP. With fewer complications, especially Grade II noted with RALP, it may be considered a better procedure for Afro-Caribbeans with prostate cancer in comparison to ORP, especially if the cancer is less severe. However, this complication profile in Afro-Caribbeans should be confirmed with a larger sample in future studies. One possible design would be to perform an age-matched case-control study comparing complication rates of ORP and RALP between non-Hispanic White patients and Afro-Caribbean patients. 

Several limitations exist for our pilot study. This is a retrospective study, the data for which was acquired from a database project in which patients that visited an academic medical center and a county hospital were entered if they met specific criteria. Since patients who had incomplete demographic data entries were excluded from our study, not all patients that potentially could have been included were captured in the database. Subjective bias may exist for information recorded by treating physicians, which could not be controlled for. The sample size is relatively small, and since all participants come from one of two sites geographically close to each other, the data may not be representative of the entire population. The sample size likely resulted in high confidence intervals for the odds ratios. In addition, the number of complications and other parameters (blood loss, surgical site infection rates, etc) that could be analyzed was limited by all that was collected in the database. Complications were considered long-term if they persisted to 18 months postoperatively, but the data did not capture any complication that may have resolved beyond this time, limiting analysis. Regardless of these limitations, this study analyzed the complication profiles for ORP and RALP in the Afro-Caribbean population. We made use of standardized complication criteria to show the lower rates of Grade II complications following RALP compared to ORP in our cohort. Nevertheless, this pilot study should be followed up with an analysis of a larger sample of Afro-Caribbean men since at the very least our study suggests a unique complication profile for this population. 

## Conclusions

Compared to ORP, RALP allows for a lower probability of complications, especially Clavien-Dindo Grade II complications, in Afro-Caribbeans. Since this differs from what previous studies suggest about the complication rates of African descent individuals when categorized into a single group, further studies should look to explore how complication rates differ in sub-ethnic groups undergoing major urologic procedures. Understanding the complication profile unique to a sub-ethnic group allows for the practice of more personalized medicine, which helps improve long-term outcomes and patient satisfaction.
